# Spectrophotometric analysis of crown discoloration following the use of silver nanoparticles combined with calcium hydroxide as intracanal medicament

**DOI:** 10.4317/jced.53743

**Published:** 2017-07-01

**Authors:** Farzaneh Afkhami, Sadaf Elahy, Alireza Mahmoudi-Nahavandi

**Affiliations:** 1 DDS, MSc, Department of Endodontics, Tehran University of Medical Sciences, International Campus, Tehran, Iran; 2 DDS, New York University, New York, US; 3 PhD, Color Imaging and Color Image Processing Department, Institute for Color Science and Technology (ICST), Tehran, Iran

## Abstract

**Background:**

Optimal antibacterial efficacy of intracanal medicaments containing silver nanoparticles (Ag-NPs) has been well documented. However, concerns remain regarding the effect of Ag-NPs on tooth color. This study aimed to assess the effects of calcium hydroxide (CH) mixed with Ag-NPs as intracanal medicaments on tooth color. The effect of location of application of medicament on the degree of discoloration was evaluated as well.

**Material and Methods:**

Fifty extracted single-rooted, single-canal human teeth with straight roots, no caries, no cracks or fractures were collected and accessed. After cleaning and shaping of the root canals, the teeth were randomly divided into two experimental groups (n=20) with CH and CH plus Ag-NPs as intracanal medicaments and a control group of saline (n=10). Experimental groups were randomly divided into two equal subgroups of A, where medicament was applied below the cemento enamel junction (CEJ) and B where the medicament was applied to the root canal and pulp chamber. Color change (ΔE) was assessed using a spectrophotometer in CIELAB system at five time points of beforemedicaments application (T0), immediately medicaments placement (T1), one week (T2), one month (T3) and three months (T4) after the application of medicaments. Data were analyzed using two-way and three-way ANOVA.

**Results:**

Color change in Ag-NPs plus CH and CH groups was not significantly different at any time point (*P*=0.23). Increased exposure time in both groups did not increase the ΔE (*P* >0.05). Significant differences were noted in ΔE between subgroups A and B (*P*<0.05).

**Conclusions:**

Addition of Ag-NPs to CH caused no significant change in tooth color compared to the application of CH alone. However, its use must be limited to the root canal space only.

** Key words:**Silver nanoparticles, color change, calcium hydroxide, spectrophotometry, intracanal medicament.

## Introduction

The goal of root canal therapy is to decrease the microbial load within the root canal system (RCS) to allow healing (1). Complete elimination of bacterial biofilm from the RCS by mechanical instrumentation alone is unlikely to occur; therefore, irrigants and medicaments are used to disinfect inaccessible areas in the complex RCS.

Recently, nanoparticles (diameter ≤100 nm) have gained popularity as antimicrobial agents as the result of their broad-spectrum antibacterial and anti-biofilm activity and biocompatibility. Their greater surface area and charge density result in their greater interaction with the bacteria. Because of the novel physical and chemical properties of nanoparticles, recent studies have focused on using them for root canal disinfection (2).

Previous studies have reported strong antibacterial and anti-biofilm efficacy of nanoparticles such as chitosan, zinc oxide, gold, silver and quaternary ammonium polyethyleneimine (2-6). Among these nanoparticles, Ag-NPs have attracted a great deal of interest due to their extensive medical applications in, wound dressings, ointments, bandages, catheters, stents and external fixation pins (7). The antibacterial efficacy of Ag-NPs in dentistry has been documented. The antibacterial effect of Ag-NPs has been shown on bacterial species such as *Staphylococcus aureus, Escherichia coli, Pseudomonas aeruginosa, Staphylococcus epidermidis* and *Enterococcus faecalis* (8-10).

Javidi *et al.* (11) and Afkhami *et al.* (12) highlighted the potential application of Ag-NPs mixed with CH as a root canal medicament; however, despite the efficacy of Ag-NPs for root canal disinfection, their possible adverse effects such as tooth discoloration made them a controversial agent for *in vivo* usage especially for long term applications as a root canal medicament (11-13).

Previous studies have shown gray-black discolorations persistent to bleaching due to the application of silver point as root canal filling material. In addition, continuous release of silver ions resulted in recurrence of discoloration. Therefore, it may be speculated that Ag-NPs may have the same effect with respect to tooth discoloration (14).

Thus the question that arised from these experimental studies is whether the addition of Ag-NPs to commonly used CH as an intracanal medicament would result in color change of tooth crown.

Considering the gap of information on tooth discoloration due to the application of Ag-NPs, this study sought to assess the effect of Ag-NPs mixed with CH on tooth color and to examine the effect of location of application of medicament in this regard.

## Material and Methods

-Tooth Preparation:

The study protocol was approved by the Ethics in Research Committee of Tehran University of Medical Sciences. Fifty recently extracted single-canal human anterior teeth with straight roots, 22±1mm length, closed apices, no caries, cracks, fractures, restorations or resorption were included in this study. Presence of a single canal was ensured radiographically. Calculus and stains were removed by a scalar followed by the use of pumice paste and a polishing cup with a low-speed hand piece. The teeth were examined for cracks and resorption under a stereomicroscope (SM X800, Nikon Co., NY, USA) and were then immersed in phosphate buffered saline (PBS) in a box away from light.

Standard straight-line access cavities were prepared using a round bur (DentsplyMaillefer, Ballaigues, Switzerland) and a fissure bur (D & Z, Wiesbaden, Germany). A #15 K-file (Dentsply, Maillefer, Ballaigues, Switzerland) was introduced into the canal until its tip was visible at the apex and the working length was determined one millimeter short of this length. Each canal was prepared using ProTaper rotary system (DentsplyMaillefer, Ballaigues, Switzerland) and Endo IT motor (VDW, Munich, Germany) according to the manufacturer’s instructions (S1, S2, F1- F5).

RC-Prep (Premier Dental Products, Norristown, PA, USA) was used as lubricant and 10 mL of 2.5% sodium hypochlorite (NaOCl) was used as irrigant during root canal preparation. A final rinse with 3mL of 17% ethylenediaminetetraacetic acid (EDTA) and 5.25% NaOCl was carried out for five minutes for smear layer removal. Each canal was then rinsed with 5 mL saline and dried with paper points. To prevent leakage of materials through the apex, the apices of all the teeth were sealed with self-cure glass ionomer (Fuji, Fuji Corporation, Japan).

A square-shaped cavity measuring 1.5×1.5 mm and 1mm in depth was prepared at the mid-center of buccal surface of all teeth with a fissure bur (DentsplyMaillefer, Ballaigues, Switzerland) to ensure standardized and reproducible measurements. Apical two-thirds of the roots were mounted in transparent acrylic resin (Acropars, Tehran, Iran) and the acrylic blocks were trimmed to have 4 cm height.

The samples were randomly assigned to three groups:

Group 1 (n=20): CH paste was applied to the root canals as an intracanal medicament (Metapex; Meta Biomed Co., Cheongiu City, Korea).

Group2 (n=20): CH paste was mixed with 100 ppm Ag-NPs suspension (NanoSany Corporation, Mashhad, Iran) in equal proportions (average nanoparticle size of 20 nm) and vortexed for one minute. This mixture was injected into the root canals using a syringe with the same tip used for Metapex.

Control group (n=10): Root canals were filled with saline.

Each experimental group was randomly divided into two subgroups of A and B. In subgroup A, the medicaments were cautiously applied only to the RCS (below the CEJ); a cotton pellet was placed into the pulp chamber and the access cavity was temporarily restored with Cavit (3M ESPE, St. Paul, MN, USA). In subgroup B, the root canals were filled with medicaments and a cotton pellet dipped in medicaments was placed in the pulp chamber and pressed to the buccal surface. The access cavity was then restored with Cavit.

Radiographs were taken to confirm optimal root canal filling in all groups. During the experiment, all the teeth were kept in a dark glass container with a piece of cotton covering them. The container incubated at 37°C and 100% humidity for three months.

-Assessment of tooth color:

Tooth color in all groups was analyzed at five time points of before (T0), immediately after (T1), one week (T2), one month (T3) and three months (T4) after the application of medicaments.

Color assessment was done in a laboratory at 20°C temperature. A piece of white non-florescent Leneta paper was used as the background as recommended by the manufacturer. Samples were fixed on a jig. Collimated light source (tungsten) illuminated the tooth surface at 45°angle relative to the vertical axis, and the spectrophotometer (CS-2000, Konica Minolta, Japan) was adjusted at 0° angle relative to the vertical axis at 70 cm distance from the tooth surface with 0.1° viewing angle. This adjustment created a circular measurement area with an approximate diameter of 1.2 mm at the center of samples. The spectrophotometer was calibrated prior to measurement of each sample and color of each sample was measured in triplicate at the center of the marked square and the mean of measured values was calculated and reported.

Color coordinates were calculated using D65/2˚viewing condition in CS-S10W software. This viewing condition is commonly used in aesthetic dentistry assays. The CIELAB color space was used for the analysis. In this color space L*, a* and b* indicate redness-greenness, b* indicates lightness, redness-greenness and yellowness-blueness respectively. Other colorimetric data including chroma (C*), hue angle (h (0 and tristimulus values were also reported by the aforementioned software. Color differences were calculated using CIE 1976 color difference formula in MATLAB software. Data acquisition was done using the CS-S10w Professional Edition software (Konica Minolta Sensing Americas, Inc., Ramsey, NJ).

**Figure 1:**
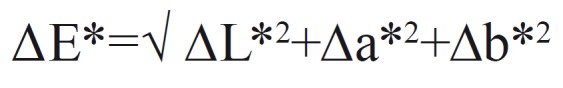
Equation.

Statistical analysis:

After obtaining the a*, L* and b* parameters, Δa*, ΔL* and Δb* were calculated. Color differences (ΔE*) was calculated using the equation below, (Fig. 1):

The ΔL* and ΔE* values, indicative of overall changes in lightness and color, respectively, were used for statistical analysis. Data were analyzed using two-way and three-way ANOVA. Post hoc ANOVA was applied whenever required. The level of statistical significance was set at 5%.

## Results

The ΔE*, ΔL*, Δb* and Δa* values for all groups are shown in  Table 1- Table 4, respectively.”.

**Table 1:**
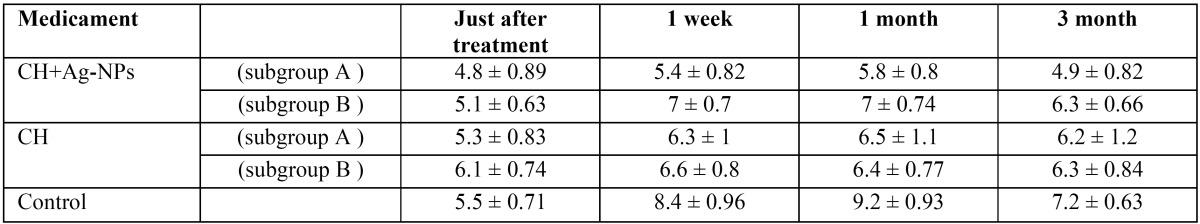
Mean ΔE* ± standard deviation of tooth samples for the different groups over the time periods.

**Table 2:**
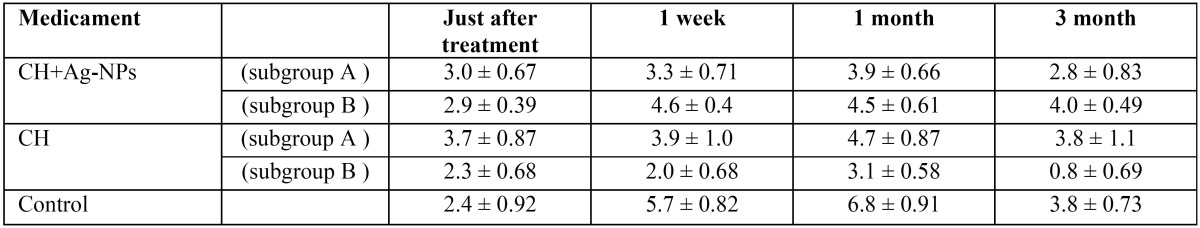
Mean ΔL* ± standard deviation of tooth samples for the different groups over the time periods.

**Table 3:**
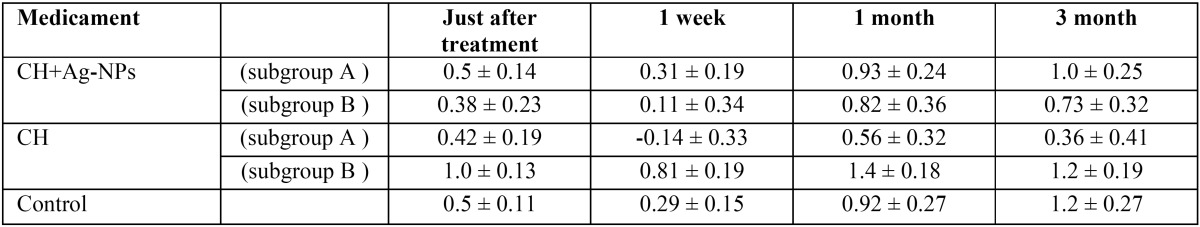
Mean Δa* ± standard deviation of tooth samples for the different groups over the time periods.

**Table 4:**
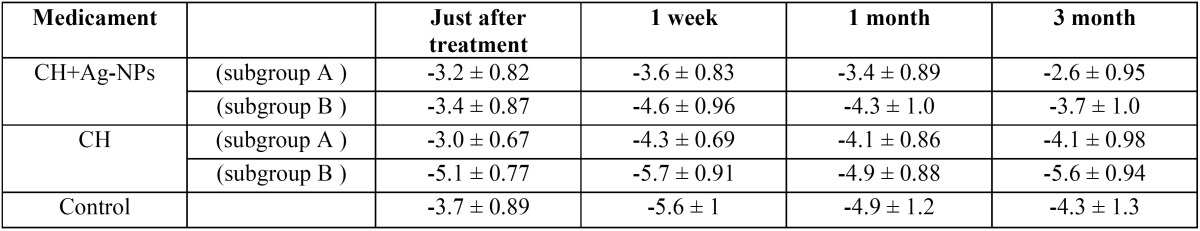
Mean Δb* ± standard deviation of tooth samples for the different groups over the time periods.

Two-way ANOVA showed that the difference in ΔE* and ΔL* values at different time points was not significant in CH group (*P*=0.82 and *P*=0.31, respectively). In CH group, a significant difference was found in ΔL* between subgroups A and B (*P*=0.001); but the difference in this regard among different time points was not significant (*P*=0.3105).

In group 2, no significant difference was noted in ΔE* among different time points (*P*=0.23); but the difference between sub-groups A and B was statistically significant (*P*=0.04). No significant difference was noted in ΔL* among different time points or between subgroups A and B (*P*=0.19 and *P*=0.08, respectively).

Significant differences were found in ΔE* and ΔL* among the three treatment groups (*P*=0.00 and *P*=0.00, respectively). Post hoc test showed a difference between the control group and the experiential groups but no significant difference was noted between the two experimental groups.

## Discussion

Tooth color can be assessed using several techniques and visual inspection is among the most commonly used techniques. Several standard methods are available for visual inspection of tooth color. In the simplest technique, the specimen and a standard shade guide or the Munsell color chips are compared by the same observer under the same standard light source. However, color perception is variable among different individuals and even in the same person at different time points; thus, this technique is associated with errors (15). In addition, the color spectrum of these shade guides is limited (16). Use of colorimetric devices seems to be more accurate. Devices used for this purpose are divided into two main groups of colorimeters, which determine the three color coordinates and are not very reliable (17) and spectrophotometers, which measure the transmitted or reflected light and determine the color parameters via the use of mathematical formulations. Color assessment by these devices eliminates the subjective errors of color analysis (18). Spectrophotometry is commonly used for measurement of transmitted or reflected light and is suitable for color analysis of convex and asymmetric objects like teeth (19). Spectrophotometers are the reference tools for color assessment and are superior to other techniques for dental applications (17). In the current study, CS2000 Konica Minolta spectrophotoradiometer was used for color assessment of tooth crowns. Also, the CIELAB color space was applied for assessment of discoloration of teeth. The L*, a*and b*parameters stand for brightness, red-green axis and yellow- blue axis, respectively (17,20). This system measures color as a numerical value and shows the overall color change or ΔE as a scalar value (17). Since the mean colorimetric coordinates at baseline are different among groups, statistical analysis of these values results in incorrect interpretation of changes. Thus, ΔL*, ΔE*, Δb* and Δa* are calculated since they are more reliable for assessment of changes at each time point; therefore, this system was used in this study.

The smear layer is composed of microcrystalline and organic debris and covers the root canal walls after root canal instrumentation. Presence of smear layer affects dentin permeability and subsequently the tooth color change. Presence of smear layer decreases tooth discoloration. Under these conditions, longer periods of time are required in order for the discoloration to occur (21). The smear layer was eliminated in the current study in order to eliminate its effect on the results and to allow better penetration of medicaments into dentinal tubules.

Endodontic medicaments are used in-between treatment sessions for necrotic teeth or for revascularization or apexification treatments. Thus, in the current study, we assessed the effects of medicaments on tooth color after variable time intervals (22,23) to assess tooth discoloration following the short-term and long-term applications of medicaments.

Calcium hydroxide is used in different forms in endodontic procedures. For greater efficacy, it may be used in conjunction with some other materials such as Ag-NPs, which have shown promising results for efficient elimination of microorganisms from the RCS (24,25). Recent evidence highlights the potential use of Ag-NPs as intracanal medicament; however, some concerns exist regarding their potential to cause tooth discoloration. This issue must be cleared prior to clinical application of this agent (26,27).

In the current study, the obtained results indicated lack of a significant difference in ΔL* and ΔE* between the two groups of CH and Ag-NPs plus CH. In other words, addition of Ag-NPs to CH caused no significant change in tooth color (neither in overall color change nor in brightness). Moreover, the results showed that long-term application of Ag-NPs plus CH as intracanal medicament caused no significant change in tooth color or brightness. However, application of intra canal medicament to the pulp chamber caused a significant change in color in comparison to the subgroup where the medicament was confined to the root canal space (subgroup A). Similarly, Kim *et al.* (21) showed tooth discoloration due to the application of medicament to the access cavity and thus, recommended that application of medicaments must be limited to the root canal space.

In all groups in our study, Δb* significantly decreased immediately after the application of medicaments, which indicates reduction in yellowness compared to baseline. Such an immediate reduction in yellowness may be attributed to the temporary restoration of access cavity with Cavit.

ΔL* in both experimental groups had significant differences with that in the control group and the teeth in experimental groups became darker. In addition, significant color change occurred in the experimental groups due to the application of medicaments compared to the control group. In a clinical trial conducted by Day *et al.*, (28) application of Ultracal XS CH medicament in replanted teeth caused significant darkening of teeth in the clinical setting; however, this color change only caused concerns in a few people. Their results were in line with our findings. Akcay *et al.* (29) reported insignificant color change in teeth due to the application of CH medicament (no significant difference with controls). Such variability in results may be attributed to different medicaments used since they applied Spotdent mixed with distilled water, which has a different composition than Metapex used in our study. Moreover, their experiment was conducted on bovine teeth; whereas, we used freshly extracted human teeth to better simulate the clinical setting.

## Conclusions

Within the limitations of this *in vitro* study, the following conclusions were drawn: Materials used as intracanal medicaments have the potential to change the tooth color. Thus, esthetic considerations must be taken into account as well as functional properties and therapeutic efficacy when selecting an intracanal medicament particularly for the anterior teeth. Addition of Ag-NPs to CH paste caused no significant change in tooth color compared to the application of CH alone. Increasing the application time of CH+Ag-NPs up to three months did not increase color change; however, application of the medicament must be confined to the root canal space and the residues in the pulp chamber must be carefully removed before restoring the crown.
